# A tiny new Middle Triassic stem-lepidosauromorph from Germany: implications for the early evolution of lepidosauromorphs and the Vellberg fauna

**DOI:** 10.1038/s41598-020-58883-x

**Published:** 2020-02-20

**Authors:** Gabriela Sobral, Tiago R. Simões, Rainer R. Schoch

**Affiliations:** 1 Staatliches Museum für Naturkunde Stuttgart, Rosenstein 1, D-70191 Stuttgart, Germany; 2 Department of Organismic and Evolutionary Biology, Museum of Comparative Zoology, Harvard University, Cambridge, MA 02138 USA

**Keywords:** Palaeontology, Taxonomy, Herpetology

## Abstract

The Middle Triassic was a time of major changes in tetrapod faunas worldwide, but the fossil record for this interval is largely obscure for terrestrial faunas. This poses a severe limitation to our understanding on the earliest stages of diversification of lineages representing some of the most diverse faunas in the world today, such as lepidosauromorphs (e.g., lizards and tuataras). Here, we report a tiny new lepidosauromorph from the Middle Triassic from Vellberg (Germany), which combines a mosaic of features from both early evolving squamates and rhynchocephalians, such as the simultaneous occurrence of a splenial bone and partial development of acrodonty. Phylogenetic analyses applying different optimality criteria, and combined morphological and molecular data, consistently recover the new taxon as a stem-lepidosauromorph, implying stem-lepidosauromorph species coinhabited areas comprising today’s central Europe at the same time as the earliest known rhynchocephalians and squamates. It further demonstrates a more complex evolutionary scenario for dental evolution in early lepidosauromorphs, with independent acquisitions of acrodonty early in their evolutionary history. The small size of most terrestrial vertebrates from Vellberg is conspicuous, contrasting to younger Triassic deposits worldwide, but comparable to Early Triassic faunas, suggesting a potential long-lasting Lilliput effect in this fauna.

## Introduction

The Middle Triassic was a time of major changes in terrestrial tetrapod faunas on a global scale. Despite the most recent divergence time estimates based on molecular and morphological data indicating the origin of most diapsid lineages during the Permian^1,2^, several lineages of diapsids are recognized in the fossil record for the first time only in the Middle Triassic, such as squamates, rhynchocephalians, tanystropheids, and drepanosaurs^2–4^. Other lineages that first appeared in the fossil record in the Early Triassic or Late Permian considerably increase in abundance and taxonomic representation during the Middle Triassic, such as ichthyosauromorphs, sauropterygians, and turtles^5–7^. Additionally, there is a generally poor vertebrate fossil record for the Early Triassic, partially owing to the relatively long period of recovery from the Permian-Triassic mass extinction^8,9^. All of those factors combined make the Middle Triassic of fundamental importance to understand the recovery of global faunas after the greatest mass extinction of the Phanerozoic, and the initial diversification of characteristic components of the modern vertebrae biota, such as archosauromorph and lepidosauromorph reptiles.

Among the tetrapod lineages that began diversifying in the fossil record at least by the Middle Triassic, lepidosauromorphs are one of the most diverse, representing one of the largest lineages of diapsid reptiles today (alongside birds), with ca. 10,500 described species^10,11^. The earliest putative stem-lepidosauromorphs first appear in the fossil record in the Late Permian—e.g. *Palaeagama*^12,13^—whereas crown lepidosauromorphs are first recognized in the Middle Triassic, represented by the oldest known squamate^2^ and rhynchocephalians^14,15^. However, the Triassic record of lepidosauromorphs is, in general, still extremely poor when compared to other lineages of diapsid reptiles^4–7^. Currently, only a few Triassic localities provide diagnostic lepidosauromorph taxa, including: the Czatkowice quarry in Poland (Early Triassic); the Vellberg locality in Germany (Middle Triassic); the Dont Formation in the Italian dolomites (Middle Triassic); the Lossiemouth Sandstone Formation in Northeast Scotland (Middle-Late Triassic); the Santa Maria Formation (Linha Bernardino locality) and Caturrita Formation (Linha São Luiz locality), in Southern Brazil (Late Triassic); and the Tytherington and Cromhall quarries in South West Britain (Late Triassic)^14,16–21^. Most of those localities bear only one or two valid lepidosauromorph species, with the most speciose of those currently represented by the collection of quarries in Southwest Britain, which include remains from six rhynchocephalian species: *Diphydontosaurus avonis*, *Planocephalosaurus robinsonae*, *Clevosaurus hudsoni*, *Clevosaurus minor*, *Clevosaurus cambrica, Clevosaurus sectumsemper*^22–26^. Therefore, most of the early fossil record of lepidosauromorphs remains largely unknown.

Here, we report a new partially articulated fossil lepidosauromorph from the Middle Triassic deposits of Vellberg in Southern Germany. The new species described here falls into the smallest size cluster so far collected from the site, and likely represents the first juvenile individual from that locality. This new taxon depicts a mosaic of features that are generally observed in both early evolving rhynchocephalians and squamates, suggesting stem-lepidosauromorphs may have survived up to the Middle Triassic. It further demonstrates a more complex scenario on dental evolutionary patterns among early lepidosaurs. Finally, this and other findings from Vellberg indicate this is one of the richest sites in the world to understand early lepidosauromorph evolution. Together with other taxa from this locality, Vellberg may also hold fundamental clues to understand the evolution of body size subsequent to the Permian-Triassic mass extinction.

## Results

Systematic palaeontology

Lepidosauromorpha Gauthier, 1984

*Vellbergia* n. g.

**Figure 1:**
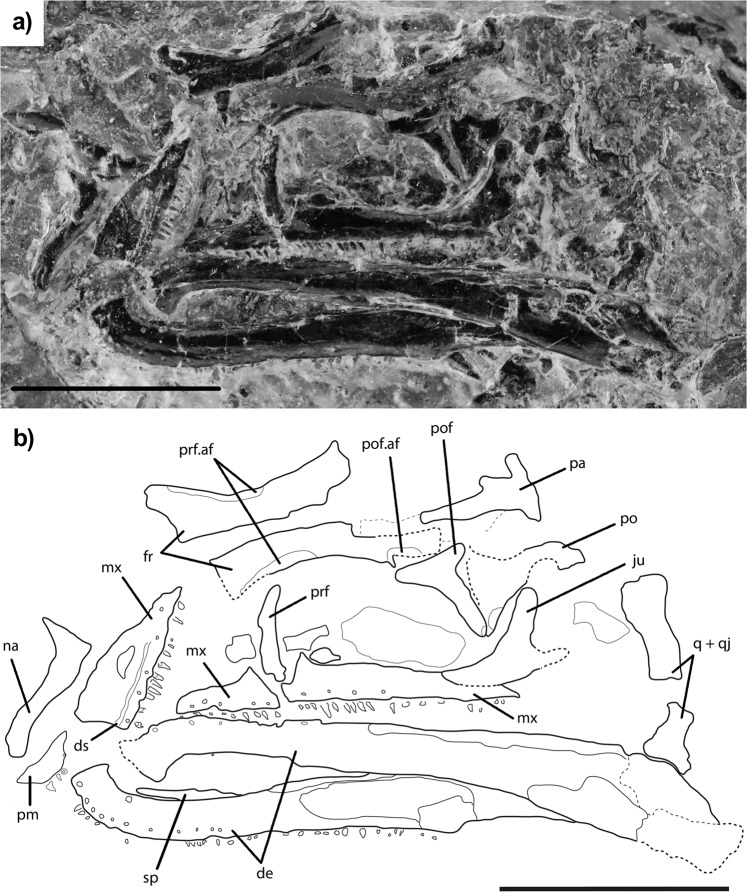
Picture (**a**) and line drawing (**b**) of the holotype material of *Vellbergia bartholomaei*. Scale bars approximately 5 mm. Abbreviations: **af** articulation facet, **de** dentary, **ds** dental shelf, **fr** frontal, **mx** maxilla, **na** nasal, **de** dentary, **ju** jugal, **pa** parietal, **pm** premaxilla, **po** postorbital, **pof** postfrontal, **prf** prefrontal, **q** quadrate, **qj** quadratojugal, **sp** splenial.

**Figure 2:**
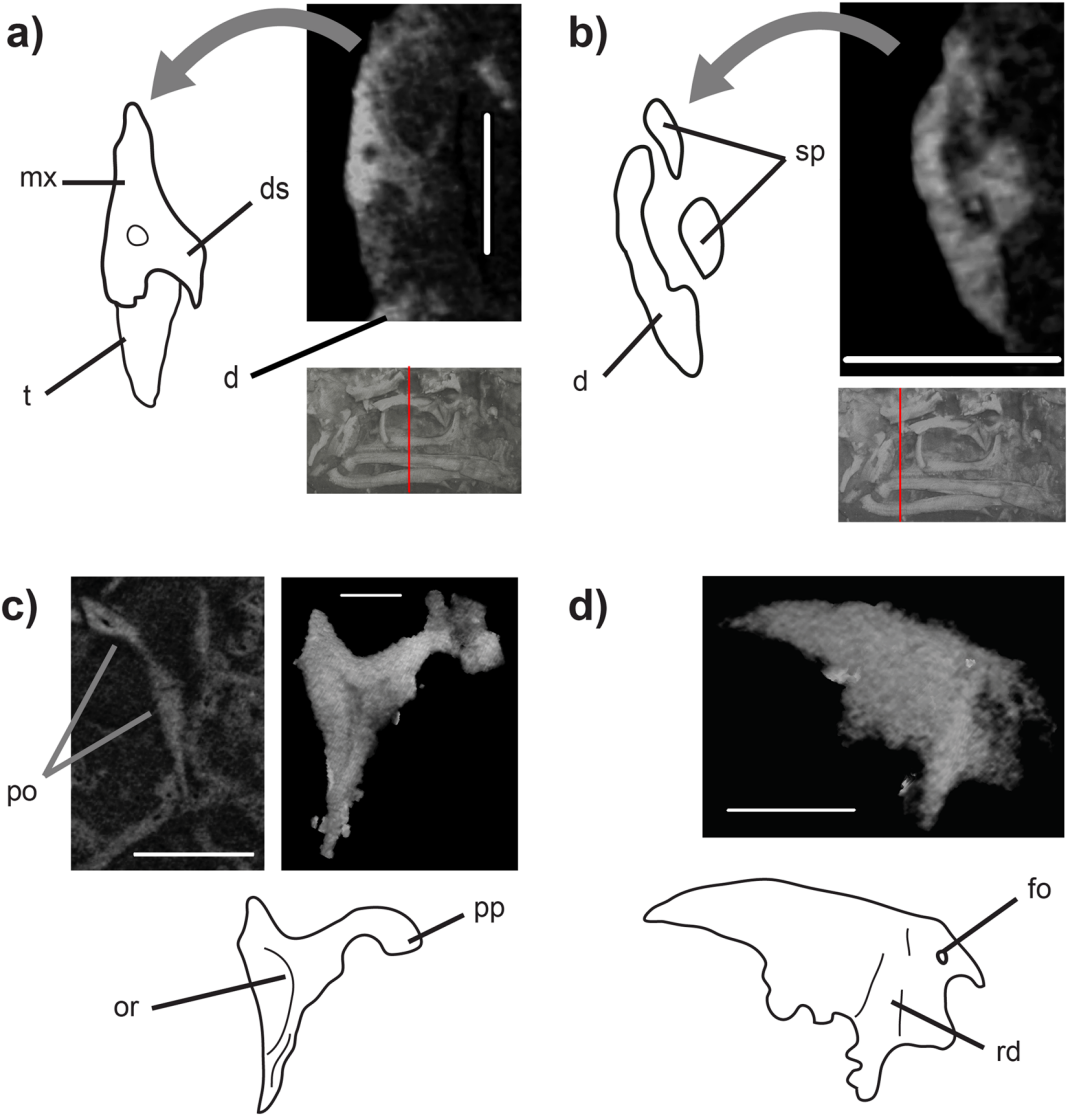
CT images of *Vellbergia*. Cross section trough (**a**) the left maxilla and (**b**) right dentary with grey miniatures below showing position of the slices. 3D renderings of the (**c**) postorbital (with CT slice) and (**d**) squamosal. Scale bars: (**a** and **b**) 0.7 mm, (**c**) 1.5 mm and 0.7 mm, (**d**) 0.95 mm. Abbreviations: **d** dentary, **ds** dental shelf, **fo** foramen, **or** orbital rim, **pp** posterior process, **rd** ridge, **sp** splenial, **t** tooth.

*Vellbergia bartholomaei* n. sp. (Figs. 1, 2, S1–S5)

**Figure 3:**
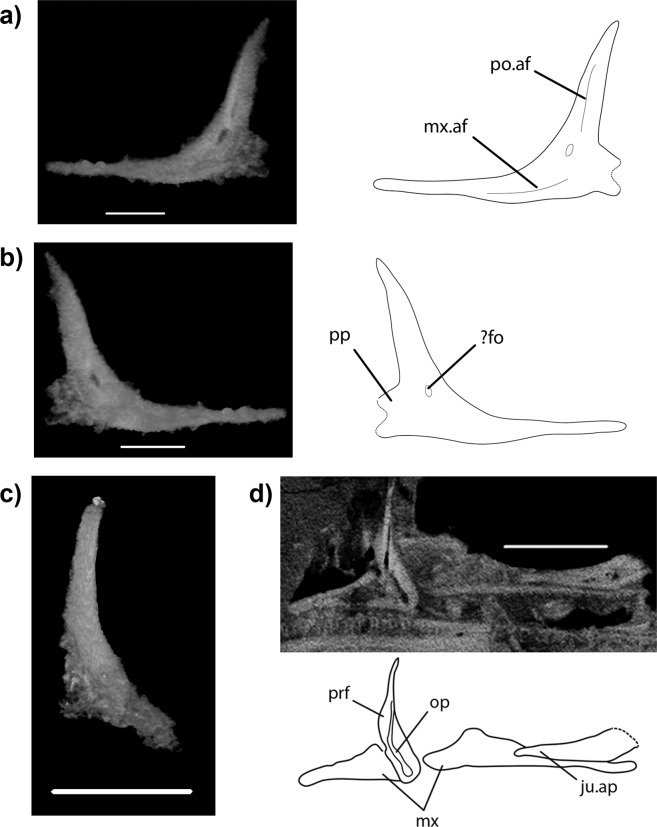
More details of *Vellbergia*. (**a**) Lateral and (**b**) medial views of the three-dimensional rendering (left) of the jugal and their corresponding line drawings (right), (**c**) left prefrontal with excess matrix removed (original segmentation in Fig. S2), and (**d**) CT scan (above) and line drawing (below) showing the relative positions of the prefrontal and the upper jaw. Scale bars: (**a** and **b**) 0.95 mm, (**c**) 2 mm, and (**d**) 2.5 mm. Abbreviations: **af** articular facet, **ap** anterior process, **fo** foramen, **ju** jugal, **mx** maxilla, **op** opening, **po** postorbital, **pp** posterior process, **prf** prefrontal.

Type: SMNS 91590, an approximately 12.5 mm long partial skull exposed in left-lateral view (Figs. 1–3; S1–S4).

Type locality: Schumann limestone quarry, Vellberg (Eschenau), Germany

Type horizon: Grey mudstone bed #6 (Schoch, 2002), Untere Graue Mergel, Lower Keuper, Middle Triassic (Ladinian).

Etymology: *Vellbergia* is named after the type locality; species name honoring Alfred Bartholomä of Neuenstein, who collected for many years in the Middle Triassic of Germany and donated much valuable material to public collections.

Diagnosis: *Vellbergia bartholomaei* is distinct from other lepidosauromorphs, including *Fraxinisaura*, by the following combination of features: symphysis strongly turned medially, T-shaped postfrontal, frontal with distinct antero-lateral and large postero-lateral processes; prefrontal dorsoventrally deep and not expanded anteriorly; small and narrow teeth, maxillary tooth row extending to posterior rim of the orbit. See Supplementary Information for full anatomical description.

## Discussion

Features of *Vellbergia*, such as the presence of well-developed subolfactory processes of the frontal, the presence of a ventrolateral process of the nasal, posterior teeth located apicolingually, and narrow, slender and short teeth relative to the lower jaw (Figs. 1–3; S1–S4), make *Vellbergia* distinct from other known lepidosauromorphs from the same locality, such as *Fraxinisaura*^27^ and a lower jaw previously attributed to *cf. Diphydontosaurus*^15^. Additionally, the relatively large size of the orbit in relation to the rest of the skull, and the overall diminutive size of the skull, suggest *Vellbergia* was not a fully grown individual. However, we disregard it as a hatchling or a young juvenile by the high degree of ossification of cranial bones like frontals and nasals. Unless *Vellbergia* had exceptionally high growth rates, even adults of this species would have comprised one of the smallest tetrapods found in the Middle Triassic fauna from Vellberg.

**Figure 4:**
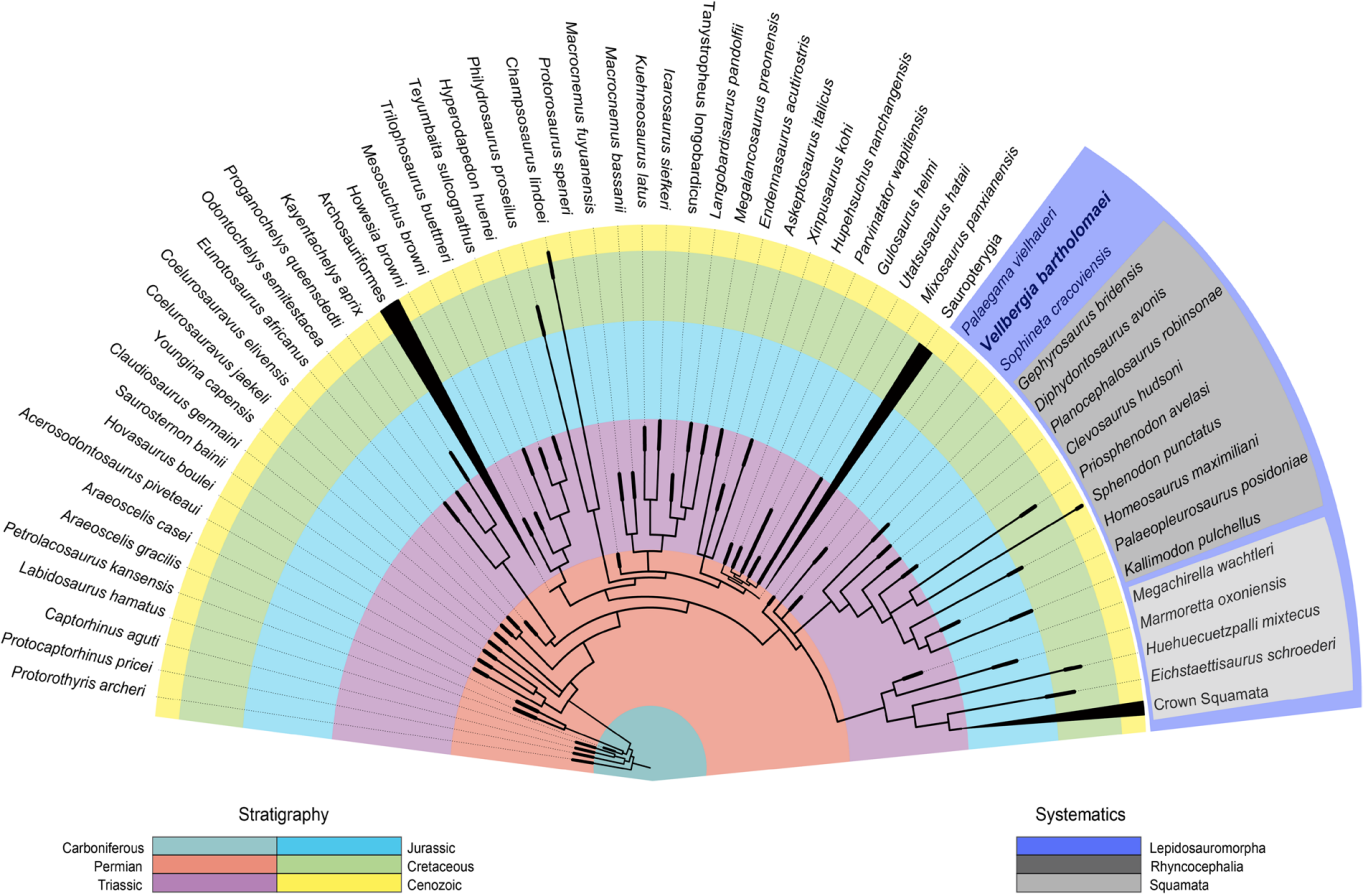
Time-scaled phylogeny depicting the relationships of *Vellbergia* based on the combined analysis of morphological and molecular data using Bayesian inference. Node positions do not reflect exact divergence times; branch lengths not to scale.

**Figure 5:**
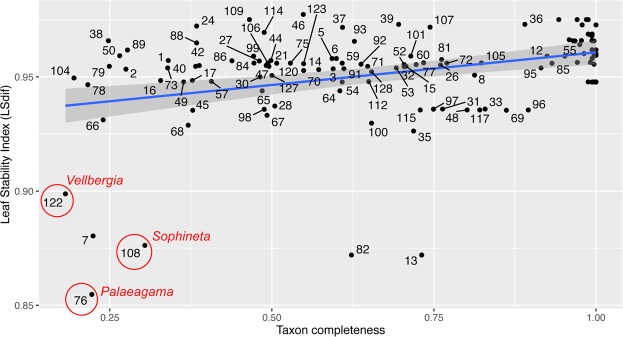
Taxon stability plotted against taxon completeness based on the posterior trees obtained from the Bayesian inference analysis. All taxa are identified in Table 1 below. Regression line in blue and 95% confidence interval in grey. Labels for extant taxa (~100% completeness) are omitted for simplicity.

In all our phylogenetic analyses, *Vellbergia* is recovered as a stem-lepidosauromorph (Figs. 4, and S7–S10). Using equal-weights maximum parsimony (EWMP), Bayesian inference (BI) with morphological data only, and BI with combined morphological and molecular data, *Vellbergia* is recovered in a polytomy with *Sophineta*^28^, rhynchocephalians, and squamates (Figs. 4, and S7, S9, S10). Maximum parsimony analysis with implied weighting (IWMP) (Fig. S8), also indicates *Vellbergia* it is a stem-lepidosauromorph, and a sister taxon to crown lepidosauromorphs (=Lepidosauria: Squamata + Rhynchocephalia), with *Sophineta* recovered as a stem-squamate. *Palaeagama*, as in previous analyses of this data set^2^, does not have a strong negative impact on tree resolution, but behaves as a rogue taxon among different analysis (Fig. 5). Whereas in parsimony-based analyses *Palaeagama* is recovered on the lineage leading to archosauriforms, in BI analyses it falls in the polytomy at the base of Lepidosauromorpha (BI-morphology only), or as the earliest evolving stem-lepidosauromorph (BI-combined data).

Overall, our results indicate *Vellbergia* is a stem-lepidosauromorph outside Rhynchocephalia and Squamata, with uncertain affinities relative to the latter two clades and *Sophineta*. Importantly, despite the holotype of *Vellbergia* being less complete than other putative early evolving lepidosauromorphs (i.e. *Sophineta* and *Palaeagama*), it is more stable in its phylogenetic placement than those other candidate stem-lepidosauromorphs (Fig. 5). Yet, given its incompleteness and the consequent reduced stability of *Vellbergia* relative to other taxa among the Bayesian posterior trees (reflected on its overall low leaf stability), we prefer to take a conservative approach and consider *Vellbergia* as a putative stem-lepidosauromorph pending the recovery of additional material.

Important morphological attributes of *Vellbergia*, most notably the elongate and slender jaw bones, the deeper post-dentary region of the jaw relative to the anterior region, and the far posteriorly reaching maxillary tooth row can be found on some other early diverging diapsid species, such as *Prolacerta* and *Youngina*^29,30^, thus showing these features were retained into the early part of the lepidosauromorph evolutionary history as well. The postorbital is found partially, and the squamosal completely, within the matrix (Fig. 2c,d). The former is typically diapsid, while the latter is very similar to *Diphydontosaurus*^24^. The jugal has a posterior process, although its full length cannot be estimated because it is incomplete (Fig. 3a,b). The prefrontal is also incomplete, lacking the anterior extension as suggested by the articular facets on the frontals (Fig. 1). The prefrontal is a ventrally elongated bone that forms the entire anterior border of the orbit. Additionally, the ventralmost portion of the prefrontal is hidden within the matrix, behind the maxilla (Fig. 3c,d). Such dorsoventrally expanded prefrontal is commonly observed among rhynchocephalians due to the absence of the lacrimal in most of those taxa, suggesting that the lacrimal was possibly absent in *Vellbergia* (but which we cannot confirm at the present).

Most importantly, however, is the contribution of the anatomical features of *Vellbergia* to our understanding of the early evolution of lepidosauromorphs. For instance, the strongly recurved symphysis of the lower jaw (Figs. 1, S1, S2, S4) is remarkably similar to early diverging rhynchocephalians, such as *Gephyrosaurus*^31^ and *Diphydontosaurus*^24^ (TRS, pers. obs.; Figs. S5, S6). Also, the dentition in *Vellbergia* has an apicolingual placement on the jaw (Fig. 2a), which is somewhat intermediate to the condition observed in the posterior teeth of *Gephyrosaurus* and *Diphydontosaurus* (resembling some of the intermediate dentary teeth in *Diphydontosaurus*). However, *Vellbergia* still retains a splenial bone (Figs. 1, 2b, S4), by which it differs from all known rhynchocephalians. Such mosaic of early lepidosaurian features provides support for its placement as a stem-lepidosauromorph and may imply a more complex evolutionary scenario for the acquisition of acrodonty in early lepidosaurs—i.e. implying the acquisition of some level of acrodonty (i.e. at least part of the dental tissue being located on the apex of the labial wall of the jaw bone^32–34^) outside rhynchocephalians among Triassic lepidosaurs. Cases of even partial development of the acrodont condition are extremely rare in non-lepidosauromorph reptiles^33^, such as in the captorhinid *Opisthodontosaurus*^32^. However, the evolution of acrodonty or pleuroacrodonty (respectively, total or partial placement of the dental tissue on the apex of the jawbone) has occurred at least five independent times in lepidosauromorphs: in sphenodontians, priscagamids, acrodontans, borioteiioids, and trogonophid amphisbaenians^33–36^. *Vellbergia* thus demonstrates that this important dental character, which is conspicuous and diagnostic for some lepidosaur lineages (e.g. sphenodontians and acrodontans in particular), was already undergoing homoplastic evolution on the earliest stages of lepidosaur evolution.

The currently available data indicate that *Vellbergia* is geologically younger than other stem-lepidosauromorphs found to date, such as *Sophineta* (Early Triassic) and *Palaeagama* (Late Permian), although the latter has less phylogenetic stability and it is sometimes recovered as more closely related to other diapsid lineages^2^ (Figs. S7, S8). With the recent phylogenetic recharacterization of *Marmoretta* (Middle Jurassic of Britain) as a stem-squamate in the most comprehensive diapsid/lepidosaur data set available to date^2^ (and herein), *Vellbergia* represents the youngest record of an early evolving lepidosauromorph. It also indicates that stem-lepidosauromorphs (*Vellbergia*), rhynchocephalians^15^ and stem-squamates^2^ occurred across islands that, today, correspond to central Europe during the Middle Triassic, with at least stem-lepidosauromorphs and rhynchocephalians cohabiting what is today South Germany.

Recent excavations in the upper Middle Triassic of Germany have revealed a plethora of new taxa, most of which comprehend small-bodied forms when compared to other tetrapod-bearing basins of this time frame. These are the deposits of Kupferzell and Vellberg, which have produced new temnospondyls^37,38^, chroniosuchians^39^, and a wide range of amniotes^17,40^. Apart from some enigmatic taxa^17^, the amniote fauna is dominated by diapsids, among which there are archosauriforms^17^, a putative choristodere, stem-turtles^5,41^ and also early evolving rhynchocephalians^15^ and one putative stem-lepidosauromorph^27^— the latter currently undergoing systematic revision. In addition to the new taxon described here and the two previously recognized lepidosauromorph species from Vellberg, two other undescribed lepidosauromorphs are known from the site (GS, RRS, in prep.), making the Vellberg locality to rival the lepidosauromorph diversity from the Late Triassic of Southwest Britain. Further, considering the higher frequency of articulated or partially articulated specimens from Vellberg (thus differing from the Polish and British Triassic localities), and its considerably older age in relation to the British deposits, we consider that the Vellberg locality represents one of the most important Triassic sites towards understanding the early evolution of lepidosauromorphs.

Besides the good representation of lepidosauromorphs, Vellberg is characterized by a large number of tetrapod fossils of small body size. Among reptiles, most species currently known for this locality are no longer than 40 mm in jaw length, even among archosauriforms (Sues *et al*.; in rev.). The exceptions are the archosauriform *Jaxtasuschus* (~80 mm skull length) and two large pseudosuchians (*Batrachotomus* and an unnamed taxon). The frequent appearance of small body sizes contrasts with younger Triassic deposits, in which different tetrapod body size classes are considerably better represented. They vary from small reptiles (e.g. lepidosaurs, parareptiles and cynodonts) with 20–50 mm in mandible/head length to archosaurs with mandible/head length and estimated body mass 10–15 times longer/larger, such as in the Santa Maria and Caturrita formations in Southern Brazil (Ladinian-Norian), Ischigualasto Formation in Northwest Argentina (Carnian–Norian), the Chinle Formation in the Southwest USA (Carnian-Rhaetian), among others^42–45^. A high representation of small-bodied faunal assemblages is more frequently observed in Triassic localities that are older than Vellberg, from Induan to the Anisian/Ladinian (first ~7–10 Myr in the Triassic). The Lilliput effect is a suggested phenomenon in which animals of small body sizes had higher rates of survival following the end-Permian, especially in lower latitudes. Smaller body sizes are capable of better heat exchange, thus providing a functional advantage during periods of fast climatic change, like the global warming around the Permian-Triassic mass extinction^9,46,47^. Such higher representation of small-bodied taxa after the Permian-Triassic mass extinction was first identified for marine invertebrates, but it has also been found more recently in terrestrial vertebrate faunas, such as in Early Triassic deposits in Russia, South Africa and Poland^16,46,47^. The low availability of Early and early Middle Triassic terrestrial deposits worldwide bearing tetrapod remains limits the assessment of body size class transitions over time. However, the location of Vellberg at a low latitude during the Ladinian (27°N)^48^, especially when compared to other known, higher-latitude contemporary localities (eg.: Santa Maria Formation, Brazil, ca. 45°S), may suggest that some low latitude localities still had a predominantly small bodied fauna by the middle Ladinian (240 Mya, or 12 Myr after the Permian-Triassic extinction), and thus a longer lasting influence of the Lilliput effect.

The observed small-bodied predominance remains to be confidently demonstrated and could, however, indicate a collecting bias instead. Small-bodied fossils require special search strategies. Since these efforts are not as commonly deployed as techniques more suitable for macrofossils, recovery of small body size diapsids is usually a by-product of finding other, more frequently targeted groups, such as archosaurs^49^. This could create a bias towards larger body sizes being sampled in most Middle to Late Triassic deposits (although this cannot explain the absence of, theoretically easier to find, large-bodied taxa in earlier Triassic strata). In any case, be it natural or human biased, concentrating collection efforts in these areas will reveal more details on the early evolution of lepidosauromorphs and other diapsid reptiles. Present-day vertebrate diversity is considerable among small-bodied animals^49^, such as lepidosaurs^11^. However, size-class bias in fossil sampling efforts, and preservation potential, currently pose as important limitations towards accurately assessing small-bodied vertebrates diversity^50^. The exploration of small-bodied vertebrates in Vellberg has already provided fundamental data towards understanding key points in the early radiation of diapsid reptiles, thus indicating we may be missing important parts of the reptile evolution by overlooking small size class materials.

## Methods

### Specimen availability

The holotype and only specimen of the new taxon is housed in the Staatliches Museum für Naturkunde Stuttgart, Germany under the number SMNS 91590. The anatomical analysis was made with the aid of computed-tomography (CT) scans performed with a Metrotom 800 Generation 1 scanner (S. Tomaschko Zeiss Computertomographie Dienstleistung, Essingen, Germany) using 110 kV and 265 µA at 500 ms and a voxel size of 17,94 µm. Segmentations and measurements were made in the software VG Studio Max 2.0 (Volume Graphics, Heidelberg, Germany). A complete anatomical description is provided in the Supplementary Material.

### Morphological and molecular data sets

In order to assess the phylogenetic placement of *Vellbergia bartholomaei* among diapsid reptiles, we included it in the recently published phylogenetic data matrix of Simões *et al*.^2^. This data set includes the largest taxonomic sampling available for early diapsid reptiles, and also includes considerable revisions on the construction of morphological characters based on discussions provided by^51,52^. This data set contains both a morphological and a molecular partition sampled for all of the extant taxa included in the data set. Owing to the beneficial effect of removing rogue taxa in phylogenetic analysis, especially in the gain in resolution in support^53^, we removed two taxa that operate as wildcards in the previous version of this data set (*Paliguana*^12,13^ and *Pamelina*^54^) [Simões *et al*.^2^, Extended Data Fig. 9]. Not all taxa identified as wildcards were removed (e.g. *Palaeagama*^12,13^ and *Sophineta*^28^), in order to find a balance between increase in tree resolution and support versus keeping a taxonomic sampling relevant to the questions addressed in the present study. Our results consist of morphology only, as well as morphological and molecular (combined evidence) analyses.

### Parsimony analysis

Analyses are conducted in TNT v. 1.1^55^ using the New Technology Search (NTS) algorithms. Tree searches are conducted using 1,000 initial trees by random addition sequences (RAS) with 100 iterations/round for each of the four NTS algorithms: Sectorial Search, Ratchet, Drift and Tree Fusing. The output trees are used as the starting trees for subsequent runs, using 1,000 iterations/rounds of each of the NTS algorithms. The latter step is repeated once again, and the final output trees are filtered for all the most parsimonious trees (MPTs).

### Bayesian inference analyses

Analyses are conducted using Mr. Bayes v. 3.2.6^56^. As there are no changes to the molecular data set we used^2^, molecular partitions and models of evolution are the same as that study. The morphological partition is analysed with the Mkv model (given that autapomorphies are included in the data set, but there are no invariable characters). Rate variation across characters is sampled from a gamma distribution, and analyses used 4 independent runs with 6 Markov chains each, sampling at every 1000 generations, for a total of 50 million generations. Convergence of independent runs is assessed using: average standard deviation of split frequencies (ASDSF ~ 0.01), potential scale reduction factors (PSRF ≈ 1 for all parameters) and effective sample size (ESS) for each parameter is greater than 200.

### Leaf stability analysis

Leaf stability was assessed using RogueNaRok^53^, which allows assessing the difference between the highest and the second highest support values for alternative resolutions of each taxon quartet/triplet in the data set (LSdif)^57^. We applied this method to the posterior trees from the Bayesian inference analysis including both morphological and molecular data. Because of the large number of taxa and large number of trees, it was necessary to downsample the total number of posterior trees from each analysis (100,000 trees after discarding burn-in). The final sample consisted of 10,000 trees (selecting one at every 10 trees) using the Burntrees script for Perl^58^.

## Supplementary information

Supplementary Information.

## Data Availability

The CT data of *Vellbergia barthomolaei*, as well as the data matrix used in the phylogenetic analyses can be accessed on Dryad under the following address: 10.5061/dryad.d2547d7zs.
