# Supplementary material for: A tiny new Middle Triassic stem-lepidosauromorph from Germany: implications for the early evolution of lepidosauromorphs and the Vellberg fauna
Source: Sci Rep. 2020 Feb 20;10:2273. doi: 10.1038/s41598-020-58883-x (PMC7033234; doi:10.1038/s41598-020-58883-x)
Supplement: Supplementary file 1 — Supplementary Information. [file 41598_2020_58883_MOESM1_ESM.pdf]

A tiny new Middle Triassic stem-lepidosauromorph from Germany: implications for the early evolution of lepidosauromorphs and the Vellberg fauna

Gabriela Sobral, Tiago R. Simões, and Rainer R. Schoch

SUPPLEMENTARY MATERIAL

1. Anatomical Description
2. Phylogenetic Analyses
3. Additional References
4. Figures
5. Table

1. Description

Skull elements are preserved mostly in articulation (Fig 1, S1); exceptions include the right maxilla, which has been detached and broken into several pieces, the right frontal located at some distance above the left frontal, the right nasal is moved anteriorly, and the right mandible turned over and situated ventrally to the left one. The postorbital region of the skull is not preserved in detail. Sutures are generally well visible, sometimes filled with grey clay matrix.

Most skull elements are smooth without traces of ornamentation. Near the dentigerous margin, there are mental foramina on the dentary and maxilla for the passage of several branches of the trigeminal nerve—terminal branches of the superior alveolar nerve in the maxilla and terminal branches of the inferior alveolar nerve in the dentary (Oelrich 1956). They are serially arranged pits near the dental margin. There are nine foramina on the preserved left maxilla and five on the right, as well as four on the left dentary and 11 on the right.

The *premaxilla* is only known from a small quadrangular fragment located at some distance

from the maxilla, preserving two straight tooth and the cross-section of a third one. It remains unclear whether the premaxilla is straight or inclined anteroventrally, or whether a premaxillary nasal process was present and how far posteriorly it could have reached.

The left *maxilla* is preserved in lateral view and in articulation with the remaining of the skull. It is broken in two pieces by the prefrontal, and the dorsal (=facial, nasal) process is damaged. It is very long, reaching as far posteriorly as the posterior end of the orbit, and forming most of the ventral margin of the orbit laterally. The right maxilla was displaced to the left side of the skull, but it is still visible in medial view, with the supradental shelf visible on the exposed surface. It shows an incomplete premaxillary ramus and a low dorsal process. The displaced right maxilla has most of the dorsal process region preserved, indicating that this process was relatively low compared to that of most diapsid reptiles. The low height of the dorsal process can also be inferred based on the low angle between the dorsal margin of the premaxillary process of the maxilla and the preserved portion of the dorsal (= facial) process of the preserved portions of the left maxilla. Additionally, the dorsal process is located at the mid-length of the maxilla. This low dorsal process of the maxilla is similar to the condition observed in early rhynchocephalians, such as *Gephyrosaurus*, but different from the early lepidosaur *Sophineta*, and the stem squamate *Megachirella* (Simões et al. 2018; TRS, pers. obs.)

The maxillae bear teeth up to the level of the main body of the jugal. It could have accommodated up to 35 teeth, and their morphology and size are rather uniform. The maxillary tooth crowns reach less than one third of the height of the maxillary ramus, and there is no maxillary lingual wall (Fig. 2A). There are no visible interdental ridges separating the teeth, although we note that this particular region is of more difficult observation due to the low resolution of the CT scans. There is no detectable ankylosis of the dental tissues to the jaw bones, and the enamel is smooth and without any signs of plicidentine.

The left *nasal* is preserved as a slender, sinuous, L-shaped element that is longer than wide, and it is preserved in dorsal view. A ventrolateral process is present at its posterior end, as also

occurring in most diapsid reptiles, extending in a right angle from the main portion of the bone. The contact with the frontal is straight. The nasal is about 30% shorter than the right frontal.

The *prefrontal* contributes to the anterior, as well as a small part of the dorsal, margins of the orbit (Fig. 1, S2a). It extended anteriorly, as indicated by its corresponding articulation facet on the frontals (Fig. 1A; prf.af), but this extension has not been preserved. The preserved part of the prefrontal is a dorsoventrally deep and gently, posteriorly curved bar of bone (Fig. 3C, D). Its ventralmost part is hidden within the matrix, medial to the maxilla and extending ventrally relative to it (Fig. 3D). An unindentified canal extends dorsoventrally within most of the bone (Figs. 3D, S2b).

The paired *frontals* are slender elements at their mid-length, and they are almost twice as wide along the anterior margin between the anterolateral processes, and about 1.5 times wider at their posterior margins. The preserved right anterolateral process is short and rounded at its anterior end, resembling in overall morphology the anterolateral processes observed in early diapsid reptiles (Schoch & Sues 2018; Sobral et al. 2015). The posterolateral ends of the frontals diverge from each other at about 20° from the midline. Articular surfaces for the pre- and postfrontal can be found on the lateral margin of the left frontal (Fig. 1b; prf.af, pof.af). The frontals formed the mid-dorsal border of the orbit. On the ventral side, the subolfactory processes (crista cranii) are more developed than in *Fraxinisaura* (Fig. S3).

The left *postfrontal* is well-preserved, but slightly displaced distally relative to the left frontal. It is an unusual element in that it is very stout and markedly T-shaped, with a ventral process that is twice as long as the other two. The angle formed between the anterior and ventral processes is slightly greater than 90°, and the angle between the ventral and posterior processes is ca. 90°. This triradiate postfrontal with a long ventral process resembles the stem- squamate *Marmoretta* (Evans 1991) and the hupehsuchian *Nanchangosaurus* (Chen et al. 2014).

The *postorbital* is mostly preserved within the matrix and not externally visible by the naked eye, thus its anatomical interpretation comes from the CT scans. It is similar to the postorbital of

many other diapsids in which the ventral process is very long and thin, whereas the dorsal process is more robust and somewhat shorter (Fig. 2C). The extent of the posterior process cannot be assessed with certainty as it is incompletely preserved, but it is robust and at least slightly longer than the ventral one. Perhaps due to the methacrylate resin applied to the specimen during its preparation, under the microscope only the facet for the jugal is visible, but segmentation of this element using CT scan data also shows a faint semilunar orbital ornamentation on the main body of the bone.

The left *jugal* is completely preserved, except for the posteriormost tip of the posteroventral process. The exposed portion of the specimen gives the impression the jugal is a short and stout element, but CT scans show that the distalmost tips of the anterior and dorsal processes lie within the matrix (Fig. 3A, B), underneath the maxilla and the postorbital, respectively. They are thus longer than they appear on the exposed portion of the matrix. The dorsal process is inclined at about 120° degrees in relation to the anterior process and measures 2,95mm (Fig. 3D). The anterior process is slightly longer than the dorsal one, reaching at about the mid-length of the orbit anteriorly. The jugal of *Vellbergia* resembles the one in other diapsid reptiles with a relatively reduced posteroventral process, such as *Pappochelys* (Schoch & Sues 2018) and *Marmoretta* (TRS. pers. obs.).

The *squamosal* is preserved within the matrix (Fig. 2D), posteriorly and medially to the postorbital. Only the process for contact with the parietal is preserved, so it is unclear whether it contains a ventral process, how long it would be or if it contacted the dorsal process of the jugal. Posterior to it, a rounded ridge indicates an elevated area which could have separated the quadrate facet from the anterior border of the bone. Posterior to this elevation, a small foramen is visible, and which we consider to not represent an artefact of preservation, as some lepidosauromorph squamosals have foramina in this area; in particular, the squamosal of *Diphydontosaurus* has a foramen in this exact position (Whiteside 1986).

In the posterior cheek region, there are two other elements that could be interpreted as being

a single broken unit representing the *quadrate* and perhaps also the *quadratojugal* (Fig. 1). The most dorsal of these elements is comprised of an articulatory head that may be missing its anteriormost portion and a long, ventrally extending shaft, which faced laterally. The preserved portion of the ventral element contains a short shaft. The base of the ventral element has a visible and well-developed mandibular condyle. CT scans show no evidence for a quadrate foramen or a quadratojugal fenestra. Further, the medial (pterygoid) process cannot be observed in the current available material. It is impossible to safely delimit which portions of the preserved elements belong to the quadrate or to the quadratojugal separately.

The *parietal*s are not entirely preserved, with only the posterior part of the right parietal being visible in ventral view and located dorsally to the squamosal. It appears T-shaped, with a modest and laterally directed supratemporal (=posterior) process. The posterior margin between the supratemporal processes is relatively straight, as also observed in the early lepidosaur *Sophineta*, many later evolving lepidosaurs, and at least some early diapsids, such as *Wumengosaurus* (Wu et al. 2011) and *Pappochelys* (Schoch & Sues 2018). The anterior end is relatively narrow and constrained laterally by the posterior ends of the frontals. The lateral margin of the parietal does not expand laterally, indicative of a relatively broad and dorsolaterally-open upper temporal fenestra. This implies on for a lateral attachment on the parietal of the external mandibular adductor musculature, which associated with a poorly developed posteroventral process of the jugal, would allow enough from for a large *M. adductor externus superficialis posterior*, as observed in most squamate lineages (Rieppel & Gronowski 1981; Simões et al. 2016).

The lower jaw is approximately 12.5mm long, with the postdentary region somewhat short. The left ramus is preserved in ventrolateral view, while the right ramus is more ventrally exposed. The posterior third of the lower jaw is twice as tall as the anterior two thirds. Unfortunately, the posterior end of the left ramus conceals the right one, precluding the observation of the details of the anatomy of the adductor and glenoid fossae. The outline of the adductor fossa, however, can be seen in the CT scans (Fig. S4).

The *dentary* bears at least 26 teeth that are similar in morphology to the maxillary ones, albeit shorter and more peg-like. Tooth implantation of the anterior dentary teeth is the same as the one observed on the maxillary dentition, with the teeth located lingually to the lateral wall of the jaw bone, with no traces of interdental ridges and no lingual wall (Fig. 2A, S4). In contrast, the posterior teeth have their bases located partially dorsally to the apex of the lateral wall of the jaw, and partially lingually to it— apicolingually located relative to the apex of the labial wall of the jaws ("pleuroacrodonty"; see main text for discussion). The dentary symphysis is strongly curved medially, a very unusual condition among early diapsid reptiles, but similar to the dentaries of some early evolving lepidosaurs, such as the rhynchocephalians *Gephyrosaurus* (Fig S5; Evans 1981) and *Diphydontosaurus* (Fig. S6, Whiteside 1986), although different from the early lepidosaur *Sophineta* (TRS pers. obs.).

The *splenial* is present. It is partially exposed on the right side of the skull, missing its anteriormost part, and also visible in the CT scans (Fig. 1, 2B, S4). It tapers at the tip and extends quite far anteriorly, approaching the mandibular symphysis and closing the Meckelian canal lingually. However, it cannot be determined if the splenial contributed to the mandibular symphysis.

2. Phylogenetic Analyses

Strict consensus tree from the equal weights maximum parsimony analysis (Fig. S7).

Best fit tree from the implied weighting maximum parsimony analysis (Fig. S8).

Majority rule consensus tree from the combined morphological and molecular data under Bayesian inference analysis (Fig. S9).

Supplementary Data: the nexus file with the full data matrix, including the scorings for *Vellbergia*.
https://datadryad.org/stash/share/NUfX5TpybkkMwkyjGGR5vPY73RJvfLzDowZgSE24pu8

3. Additional References

Chen X-H, Motani R, Cheng L, Jiang D-Y & Rieppel O. 2014. The enigmatic marine reptile *Nanchangosaurus* from the Lower Triassic of Hubei, China and the phylogenetic affinities of Hupehsuchia. PLoS ONE 9: e102361. doi:10.1371/journal.pone.0102361

Evans SE. 1980. The skull of a new eosuchian reptile from the Lower Jurassic of South Wales. Zool. J. Linn. Soc. 70, 203-264. (doi:10.1111/j.1096-3642.1980.tb00852.x).

Evans SE. 1991. A new lizard-like reptile (Diapsida: Lepidosauromorpha) from the Middle Jurassic of England. Zool. J. Linn. Soc. 103, 391-412. (https://doi.org/10.1111/j.1096-3642.1991.tb00910.x).

Oelrich TM. 1956. The Anatomy of the head of *Ctenosaura pectinata* (Iguanidae). Miscellaneous Publications, 94.

Rieppel, O, Gronowski, RW. 1981 The loss of the lower temporal arcade in diapsid reptiles. Zool. J. Linn. Soc. 72, 203-217. (doi:10.1111/j.1096-3642.1981.tb01570.x).

Schoch RR & Sues H-D. 2018. Osteology of the Middle Triassic stem-turtle *Pappochelys rosinae* and the early evolution of the turtle skeleton. Journal of Systematic Palaeontology, 16:927–965 https://doi.org/10.1080/14772019.2017.1354936.

Simões, TR, Funston, GF, Vafaeian, B, Nydam, RL, Doschak, MR, Caldwell, MW. 2016 Reacquisition of the lower temporal bar in sexually dimorphic fossil lizards provides a rare case of convergent evolution. Sci. Rep. 6, 24087. (doi: 10.1038/srep24087).

Simões TR, Caldwell MW, Tałanda M, Bernardi M, Palci A, Vernygora O, Bernardini F, Mancini L & Nydam RL. 2018. The origin of squamates revealed by a Middle Triassic lizard from the Italian Alps. Nature 557:706–709.

Sobral G, Sues H-D & Müller J. 2015. Anatomy of the enigmatic reptile *Elachistosuchus huenei* Janensch, 1949 (Reptilia: Diapsida) from the Upper Triassic of Germany and its relevance for the origin of Sauria. PLoS ONE 10: e0135114. doi:10.1371/journal.pone.0135114

Whiteside DI. 1986. The head skeleton of the Rhaetian sphenodontid Diphydontosaurus avonis gen. et sp. nov. and the modernizing of a living fossil. Phil. Trans. R. Soc. Lond. B 312, 379-430. (doi: 10.1098/rstb.1986.0014)

Wu X-C, Cheng Y-N, Li C, Zhao L-J & Sato T. 2011. New information on *Wumengosaurus delicatomandibularis* Jiang et al., 2008 (Diapsida: Sauropterygia), with a revision of the osteology and phylogeny of the taxon. Journal of Vertebrate Paleontology 31:70–83.

4. Figures

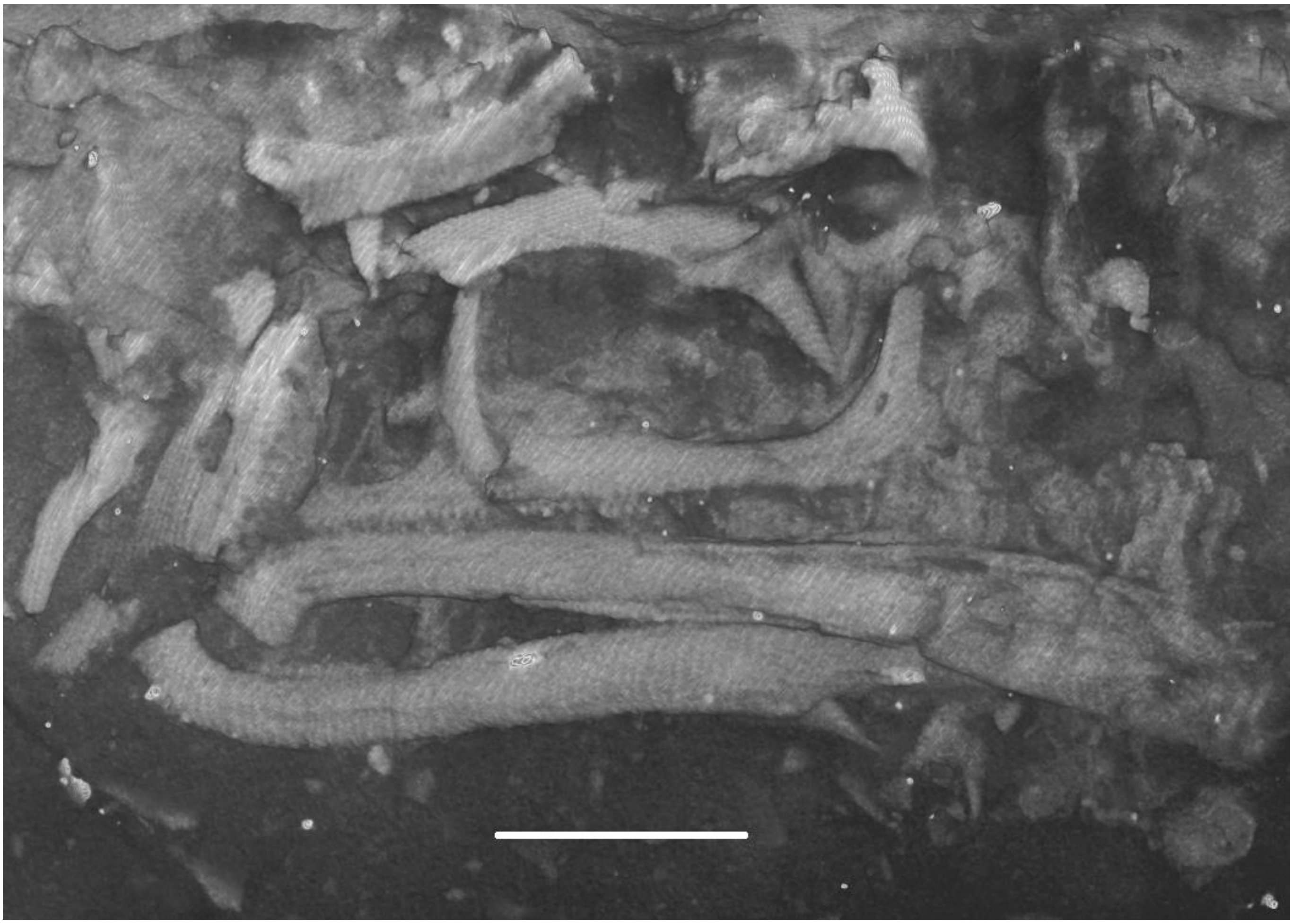

Figure S1. 3D rendering of the CT scans of *Vellbergia bartholomaei* (SMNS 91590). Scale bar equals 3mm.

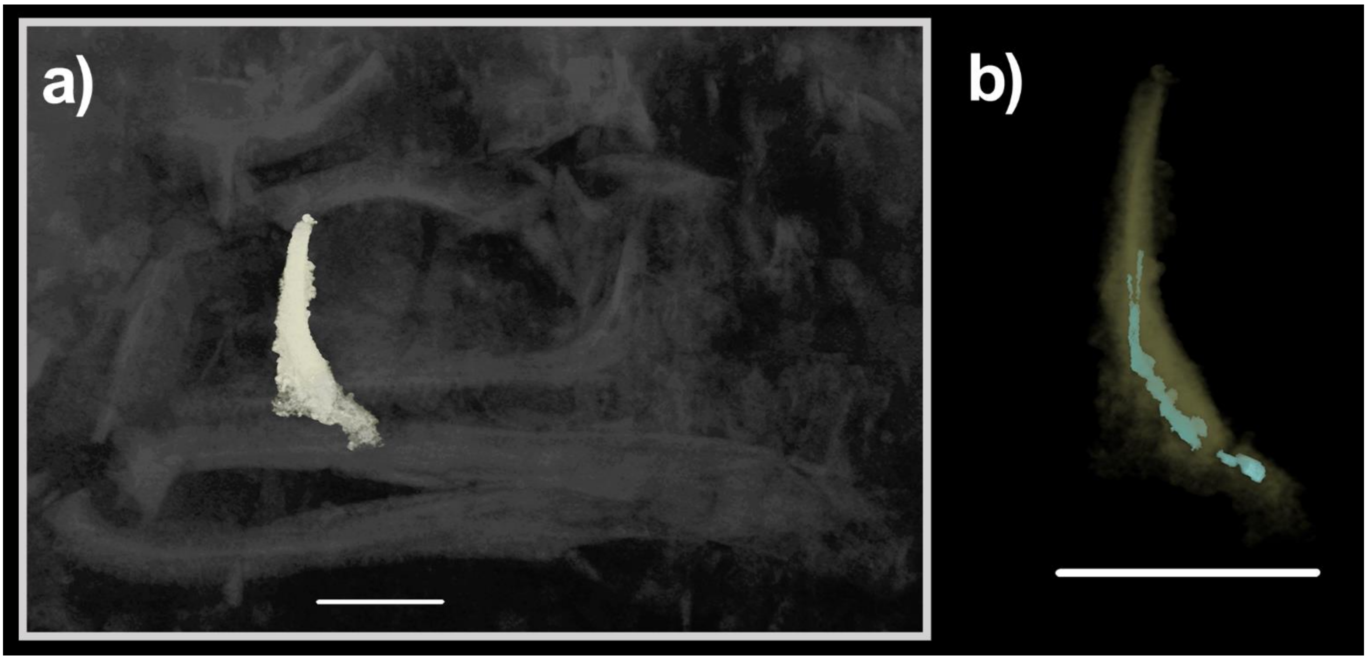

Figure S2. CT scans of the prefrontal. A) 3D rendering of the bone showing its position relative to other skull elements (semi-transparent); B) semi-transparent 3D rendering showing opening within the bone. Scale bars equal 2mm.

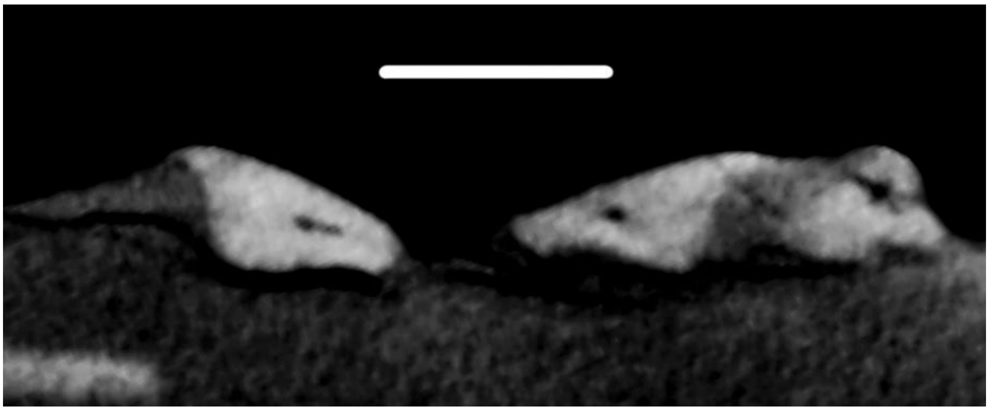

Figure S3. Cross section at about the midlength the frontals in posterior view to show the crista cranii (=subolfactory process; cc) on the ventral surface of the bones. Scale bar equals 0,75mm.

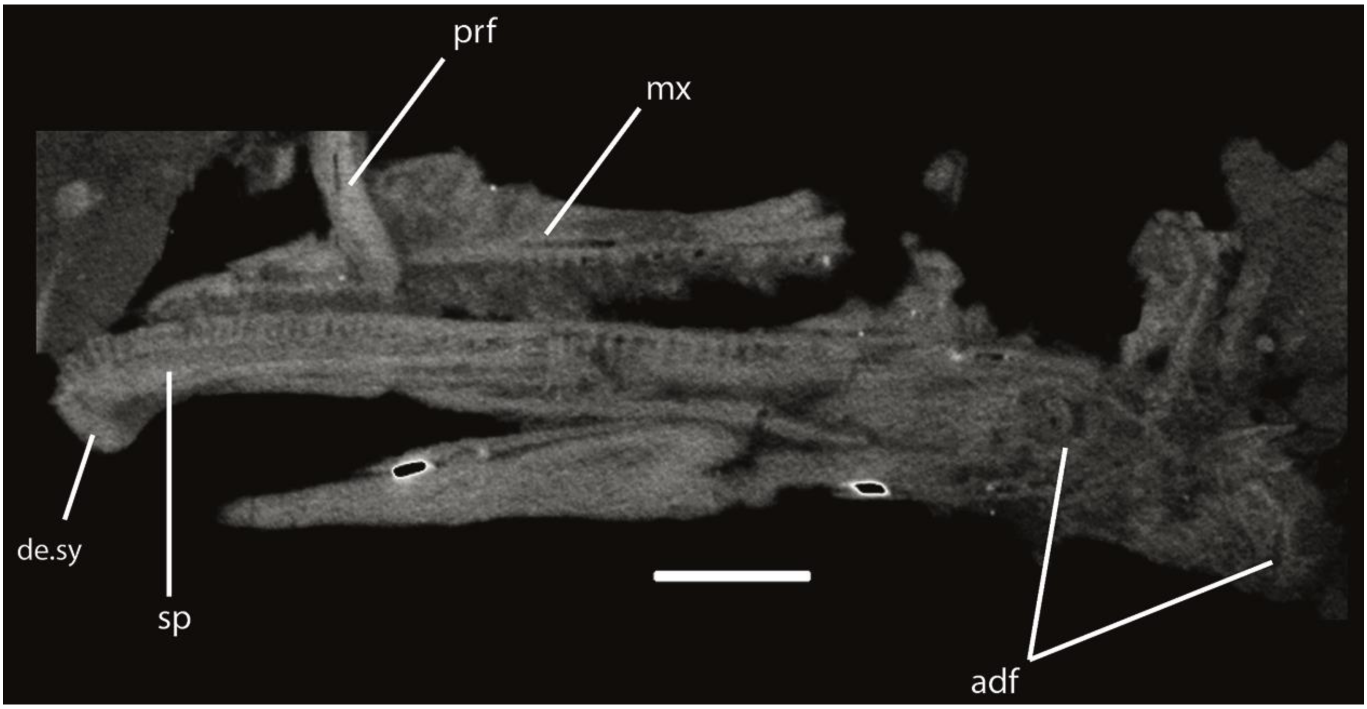

Figure S4. Longitudinal slice through the left lower jaw to show the outline of the adductor fossa (**adf**). The splenial (**sp**) and the dentary symphysis (**de.sy**) are also visible at the anterior end. Scale bar equals 1.5mm. Further abbreviaitons: **prf** prefrontal, **mx** maxilla.

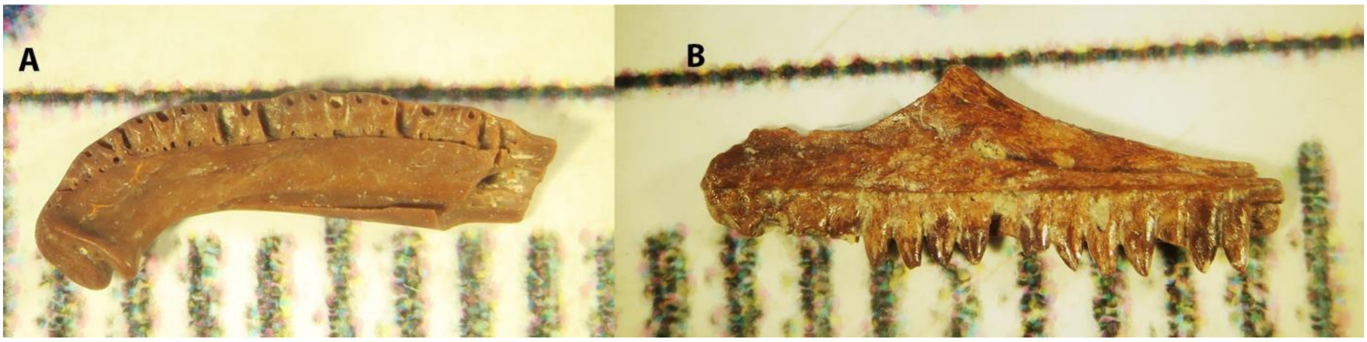

Fig. S5. A. Medially curved dentary symphysis of *Gephyrosaurus bridensis* (NHMUK RU. T. 1512). B, maxilla with low facial process of *Gephyrosaurus bridensis* (NHMUK RU. T. 1015). Scale bar in mm.

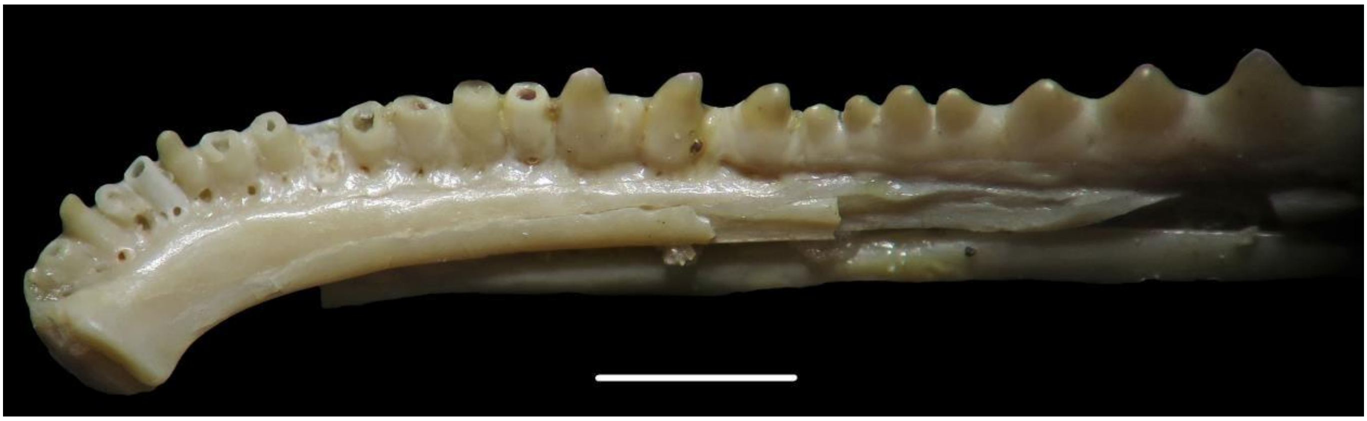

Figure S6. Photo of the right dentary of the holotype of *Diphydontosaurus avonis* (BU 23760) showing the strongly medially recurved symphysis, and the transition between pleurodont (anteriorly) and acrodont (posteriorly) teeth in the lower jaw for. Scale bar equals 1mm.

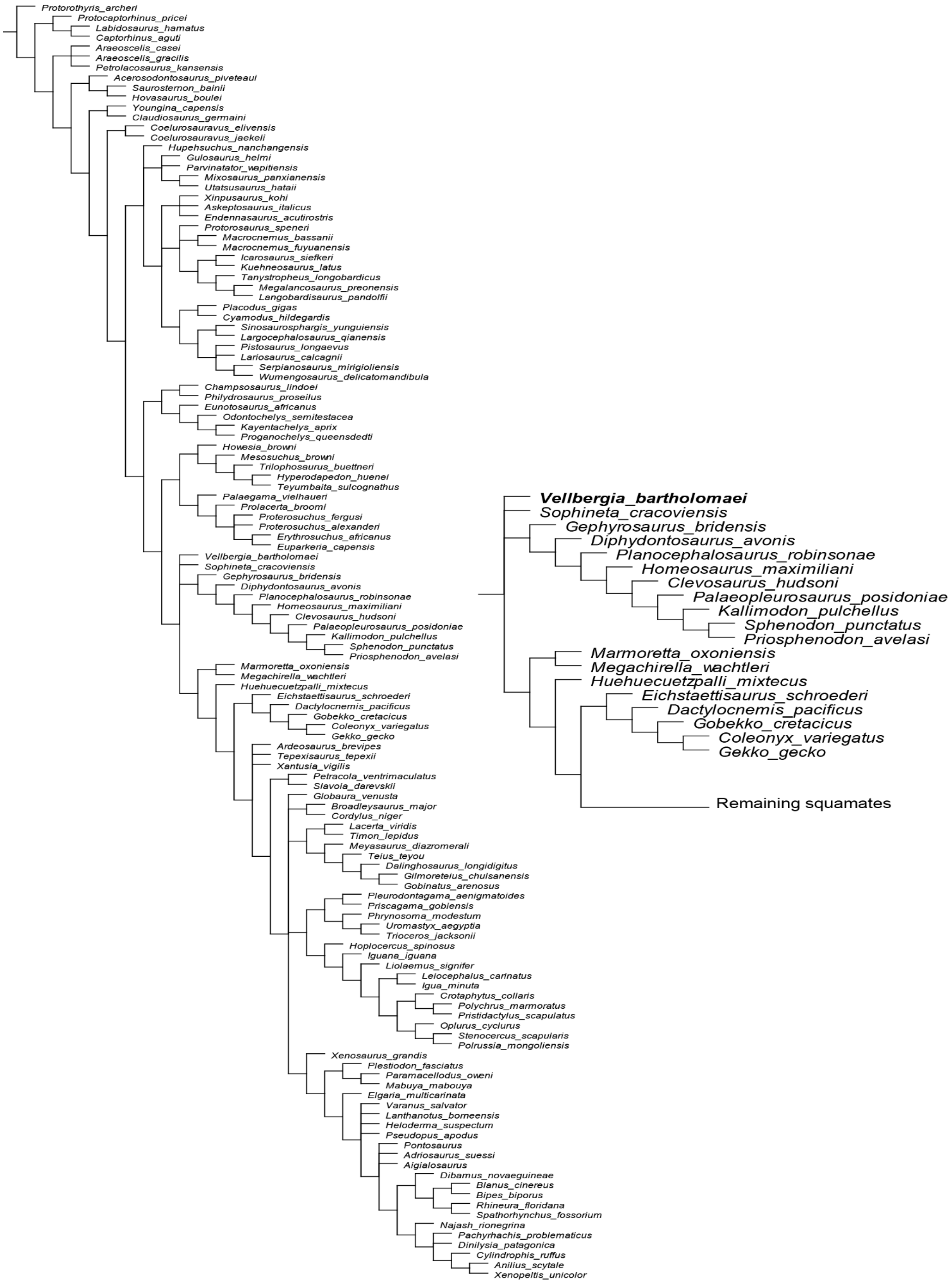

Figure S7. Strict consensus of 1027 most parsimonious trees of 2270 steps each from the equal-weights maximum parsimony analysis, with the relationships of early lepidosauromorphs in detail.

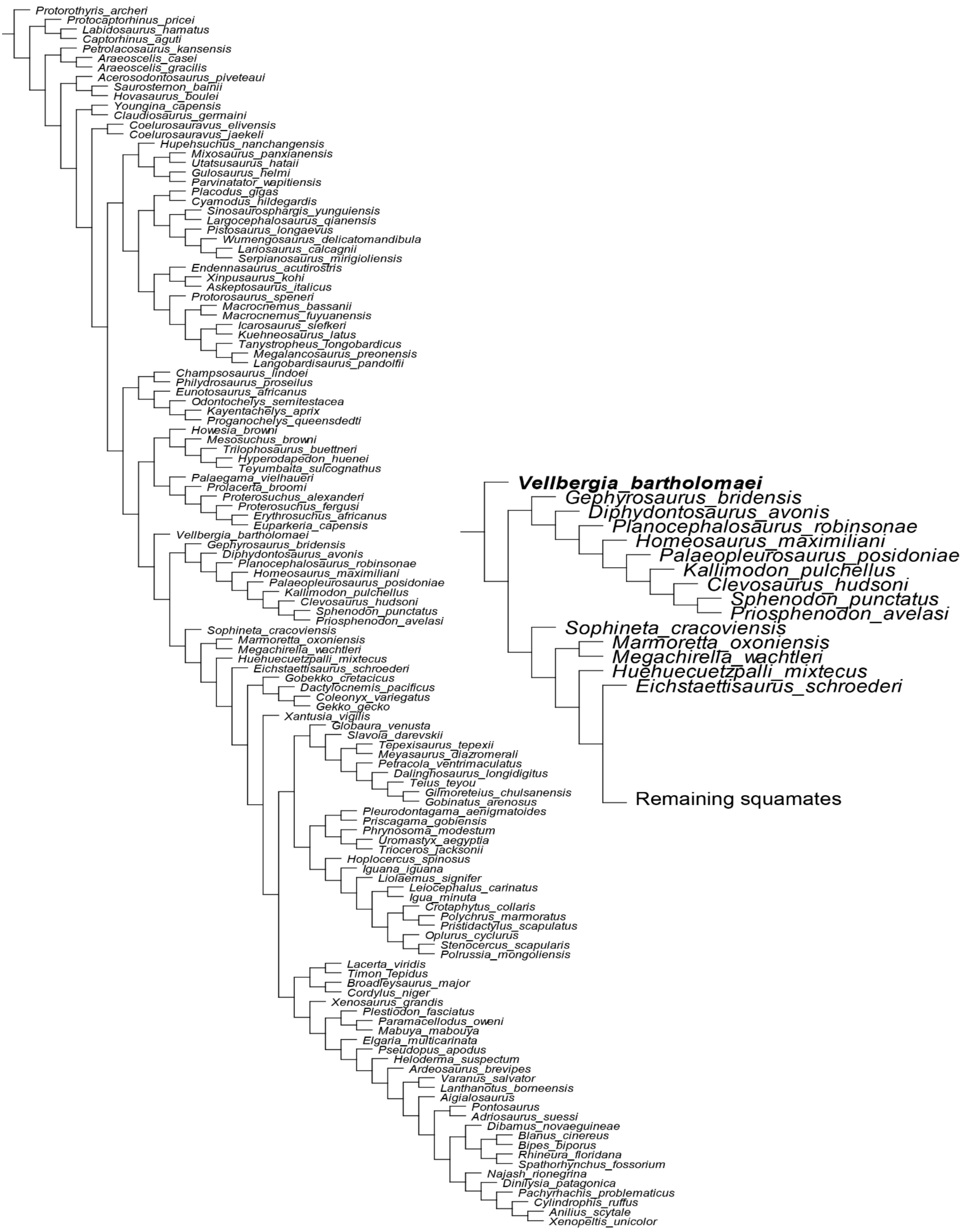

Figure S8. Single best fit tree (fit=91.881431) from the implied-weighting maximum parsimony analysis, with relationships of early lepidosauromorphs in detail.

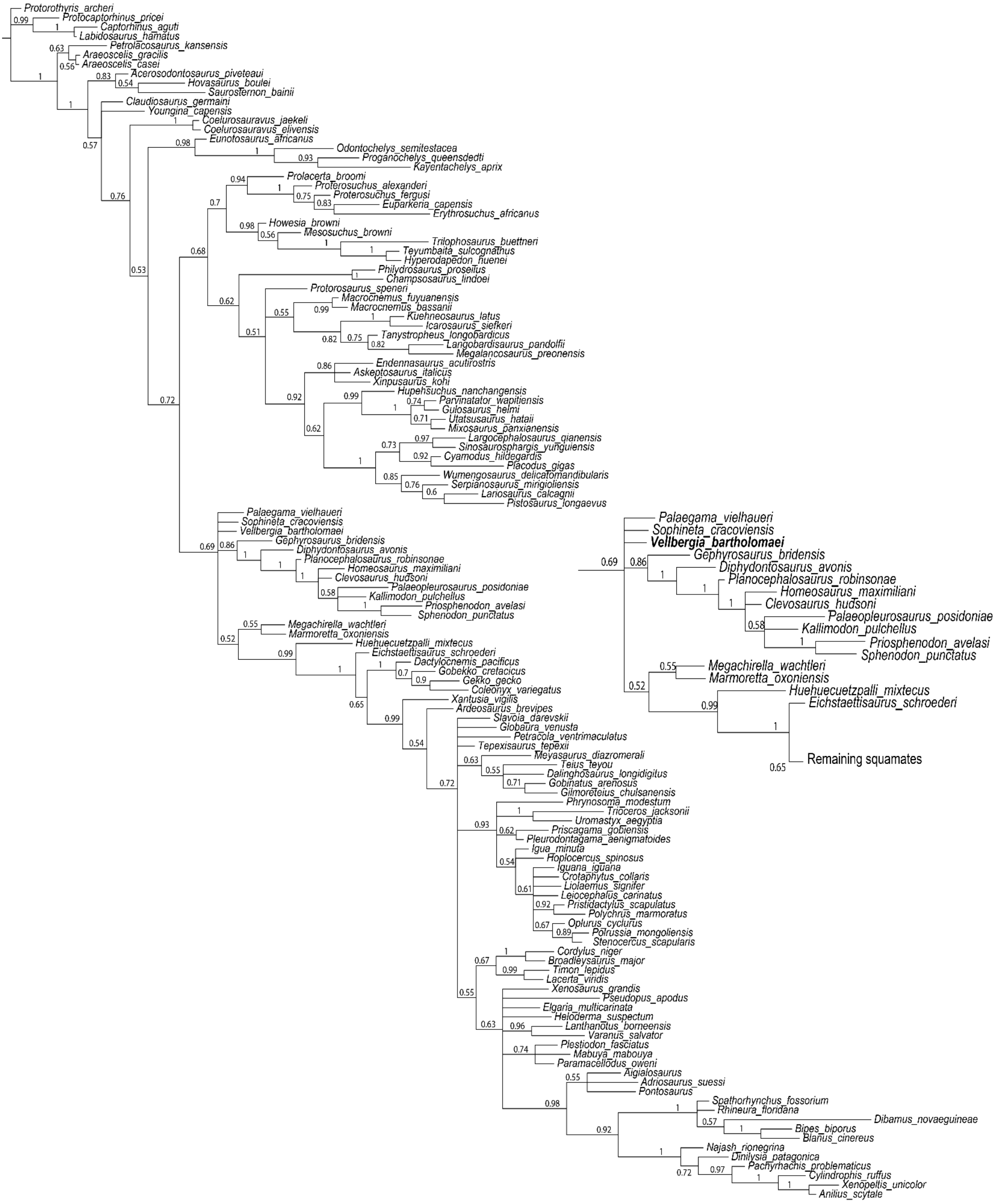

Figure S9. Majority rule consensus tree of the Bayesian inference analysis of the morphological data only, with the relationships of early lepidosauromorphs in detail. Node numbers indicate posterior probabilities.

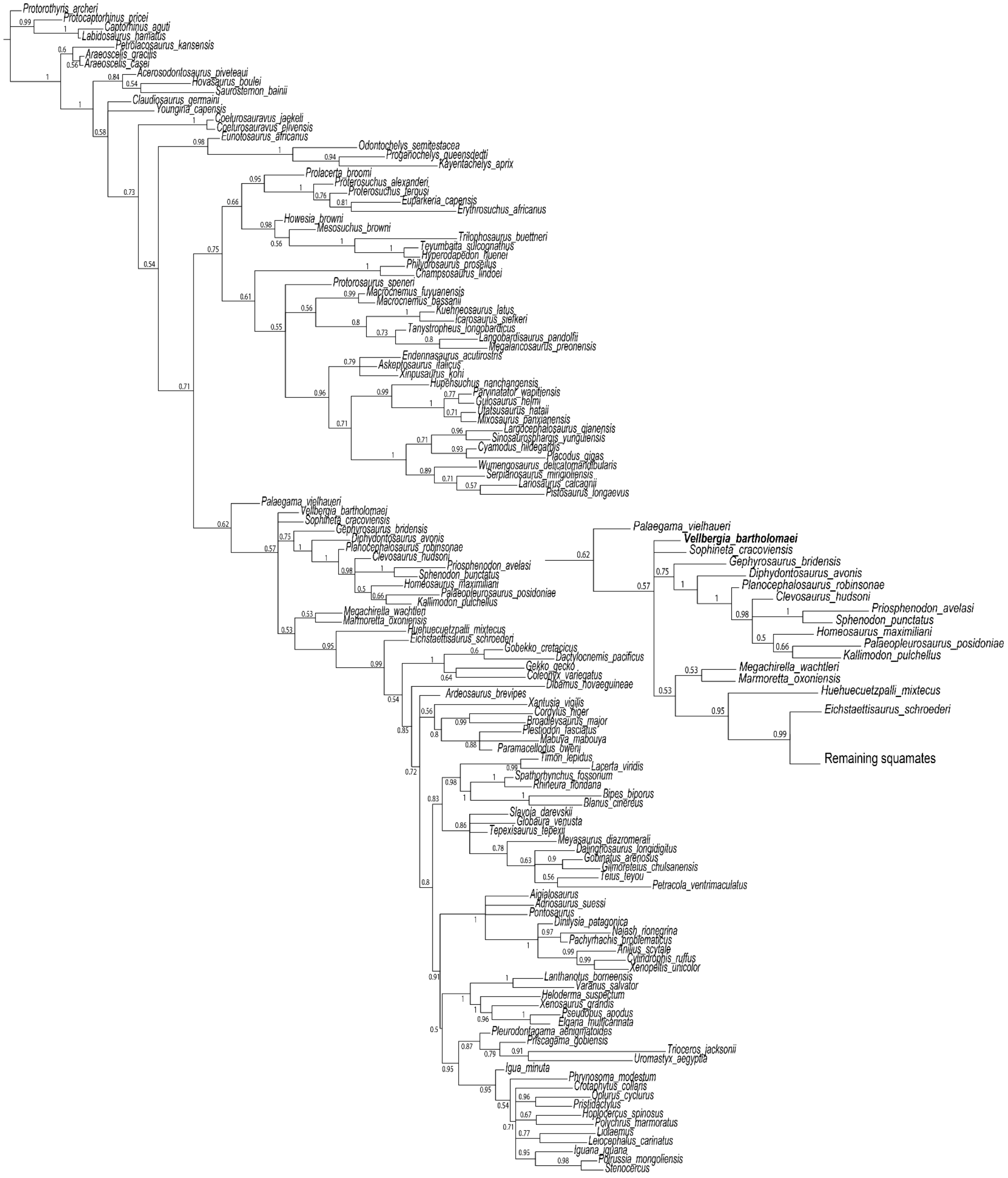

Figure S10. Majority rule consensus tree of the Bayesian inference analysis of the combined morphological and molecular data, with the relationships of early lepidosauromorphs in detail. Node numbers indicate posterior probabilities.

5. Tables.

Table 1. List of taxa for leaf stability index (lsDif) analysis depicted in Fig. 5.

Number	Taxon	lsDif_	Missing Data	Completeness
1	*Acerosodontosaurus_piveteaui*	0.957181	0.659824	0.340176
2	*Adriosaurus_suessi*	0.953393	0.725146	0.274854
3	*Aigialosaurus*	0.953534	0.405488	0.594512
4	*Anilius_scytale*	0.956359	0.028807	0.971193
5	*Araeoscelis_casei*	0.958053	0.40708	0.59292
6	*Araeoscelis_gracilis*	0.958072	0.400593	0.599407
7	*Ardeosaurus_brevipes*	0.880354	0.775811	0.224189
8	*Askeptosaurus_italicus*	0.950789	0.187311	0.812689
9	*Bipes_biporus*	0.975116	0.01487	0.98513
10	*Blanus_cinereus*	0.975116	0.057377	0.942623
11	*Broadleysaurus_major*	0.968773	0.00597	0.99403
12	*Captorhinus_aguti*	0.959119	0.075529	0.924471
13	*Champsosaurus_lindoei*	0.872004	0.268882	0.731118
14	*Claudiosaurus_germaini*	0.953219	0.427729	0.572271
15	*Clevosaurus_hudsoni*	0.954644	0.293051	0.706949
16	*Coelurosauravus_elivensis*	0.948424	0.671642	0.328358
17	*Coelurosauravus_jaekeli*	0.948424	0.622754	0.377246
18	*Coleonyx_variegatus*	0.965937	0.022508	0.977492
19	*Cordylus_niger*	0.968773	0.011976	0.988024
20	*Crotaphytus_collaris*	0.959444	0	1
21	*Cyamodus_hildegardis*	0.956004	0.492582	0.507418
22	*Cylindrophis_ruffus*	0.956362	0.028807	0.971193

23	*Dactylocnemis_pacificus*	0.965833	0.035144	0.964856
24	*Dalinghosaurus_longidigitus*	0.972287	0.616071	0.383929
25	*Dibamus_novaeguineae*	0.966288	0.041322	0.958678
26	*Dinilysia_patagonica*	0.9552	0.239852	0.760148
27	*Diphydontosaurus_avonis*	0.956977	0.519288	0.480712
28	*Eichstaettisaurus_schroederi*	0.937251	0.495468	0.504532
29	*Elgaria_multicarinata*	0.947795	0	1
30	*Endennasaurus_acutirostris*	0.949959	0.517964	0.482036
31	*Erythrosuchus_africanus*	0.935949	0.236686	0.763314
32	*Eunotosaurus_africanus*	0.954461	0.295858	0.704142
33	*Euparkeria_capensis*	0.935941	0.171091	0.828909
34	*Gekko_gecko*	0.965923	0.00639	0.99361
35	*Gephyrosaurus_bridensis*	0.926295	0.281437	0.718563
36	*Gilmoreteius_chulsanensis*	0.973021	0.109756	0.890244
37	*Globaura_venusta*	0.97171	0.391045	0.608955
38	*Gobekko_cretacicus*	0.965862	0.752239	0.247761
39	*Gobinatus_arenosus*	0.973014	0.304478	0.695522
40	*Gulosaurus_helmi*	0.95465	0.617647	0.382353
41	*Heloderma_suspectum*	0.947761	0.006154	0.993846
42	*Homeosaurus_maximiliani*	0.954993	0.61194	0.38806
43	*Hoplocercus_spinosus*	0.958078	0.006042	0.993958
44	*Hovasaurus_boulei*	0.957183	0.501466	0.498534
45	*Howesia_browni*	0.935394	0.622093	0.377907

46	*Huehuecuetzpalli_mixtecus*	0.977377	0.451515	0.548485
47	*Hupehsuchus_nanchangensis*	0.955018	0.508772	0.491228
48	*Hyperodapedon_huenei*	0.935522	0.198795	0.801205
49	*Icarosaurus_siefkeri*	0.947792	0.636095	0.363905
50	*Igua_minuta*	0.959275	0.734694	0.265306
51	*Iguana_iguana*	0.961863	0	1
52	*Kallimodon_pulchellus*	0.955402	0.296073	0.703927
53	*Kayentachelys_aprix*	0.953954	0.308197	0.691803
54	*Kuehneosaurus_latus*	0.947792	0.391566	0.608434
55	*Labidosaurus_hamatus*	0.959119	0.048193	0.951807
56	*Lacerta_viridis*	0.975081	0.012232	0.987768
57	*Langobardisaurus_pandolfii*	0.948052	0.593272	0.406728
58	*Lanthanotus_borneensis*	0.947825	0	1
59	*Largocephalosaurus_qianensis*	0.95603	0.391566	0.608434
60	*Lariosaurus_calcagnii*	0.956163	0.265861	0.734139
61	*Leiocephalus_carinatus*	0.959884	0.003012	0.996988
62	*Liolaemus*	0.96009	0	1
63	*Mabuya_mabouya*	0.968513	0.009202	0.990798
64	*Macrocnemus_bassanii*	0.943904	0.395137	0.604863
65	*Macrocnemus_fuyuanensis*	0.943904	0.51497	0.48503
66	*Marmoretta_oxoniensis*	0.931171	0.760234	0.239766
67	*Megachirella_wachtleri*	0.9332	0.507463	0.492537
68	*Megalancosaurus_preonensis*	0.928861	0.62908	0.37092
69	*Mesosuchus_browni*	0.935409	0.138235	0.861765
70	*Meyasaurus_diazromerali*	0.95281	0.451039	0.548961

71	*Mixosaurus_panxianensis*	0.954602	0.352239	0.647761
72	*Najash_rionegrina*	0.955993	0.230216	0.769784
73	*Odontochelys_semitestacea*	0.953954	0.660767	0.339233
74	*Oplurus_cyclurus*	0.960232	0.018405	0.981595
75	*Pachyrhachis_problematicus*	0.95603	0.471429	0.528571
76	*Palaegama_vielhaueri*	0.854735	0.777778	0.222222
77	*Palaeopleurosaurus_posidoniae*	0.955413	0.277439	0.722561
78	*Paramacellodus_oweni*	0.946591	0.783862	0.216138
79	*Parvinatator_wapitiensis*	0.954648	0.750725	0.249275
80	*Petracola_ventrimaculatus*	0.972783	0.021407	0.978593
81	*Petrolacosaurus_kansensis*	0.957605	0.238095	0.761905
82	*Philydrosaurus_proseilus*	0.872004	0.377246	0.622754
83	*Phrynosoma_modestum*	0.964138	0.025	0.975
84	*Pistosaurus_longaevus*	0.956082	0.527607	0.472393
85	*Placodus_gigas*	0.955981	0.067901	0.932099
86	*Planocephalosaurus_robinsonae*	0.957067	0.561562	0.438438
87	*Plestiodon_fasciatus*	0.968517	0.006154	0.993846
88	*Pleurodontagama_aenigmatoides*	0.964906	0.616071	0.383929
89	*Polrussia_mongoliensis*	0.961773	0.722714	0.277286
90	*Polychrus_marmoratus*	0.959411	0.003049	0.996951
91	*Pontosaurus*	0.953548	0.388889	0.611111
92	*Priosphenodon_avelasi*	0.955571	0.3625	0.6375

93	*Priscagama_gobiensis*	0.965592	0.372671	0.627329
94	*Pristidactylus*	0.960232	0.003021	0.996979
95	*Proganochelys_queensdedti*	0.953954	0.084848	0.915152
96	*Prolacerta_broomi*	0.935533	0.104478	0.895522
97	*Proterosuchus_alexanderi*	0.935893	0.250737	0.749263
98	*Proterosuchus_fergusi*	0.935828	0.511765	0.488235
99	*Protocaptorhinus_pricei*	0.959114	0.52819	0.47181
100	*Protorosaurus_speneri*	0.929748	0.346269	0.653731
101	*Protorothyris_archeri*	0.959114	0.285714	0.714286
102	*Pseudopus_apodus*	0.947795	0.003472	0.996528
103	*Rhineura_floridana*	0.975116	0.029167	0.970833
104	*Saurosternon_bainii*	0.949533	0.805233	0.194767
105	*Serpianosaurus_mirigioliensis*	0.956102	0.176829	0.823171
106	*Sinosaurosphargis_yunguiensis*	0.956025	0.506135	0.493865
107	*Slavoia_darevskii*	0.971796	0.255952	0.744048
108	*Sophineta_cracoviensis*	0.876217	0.695906	0.304094
109	*Spathorhynchus_fossorium*	0.975116	0.534591	0.465409
110	*Sphenodon_punctatus*	0.955571	0.003086	0.996914
111	*Stenocercus*	0.961883	0.006098	0.993902
112	*Tanystropheus_longobardicus*	0.947966	0.35061	0.64939
113	*Teius_teyou*	0.972766	0	1
114	*Tepexisaurus_tepexii*	0.969477	0.511834	0.488166
115	*Teyumbaita_sulcognathus*	0.935522	0.271084	0.728916
116	*Timon_lepidus*	0.975081	0.012085	0.987915
117	*Trilophosaurus_buettneri*	0.935521	0.179331	0.820669

118	*Trioceros_jacksonii*	0.965747	0.032362	0.967638
119	*Uromastyx_aegyptia*	0.965746	0.003077	0.996923
120	*Utatsusaurus_hataii*	0.954605	0.504478	0.495522
121	*Varanus_salvator*	0.947825	0.012158	0.987842
122	*Vellbergia_bartholomaei*	0.898792	0.818444	0.181556
123	*Wumengosaurus_delicatomandibularis*	0.955782	0.451039	0.548961
124	*Xantusia_vigilis*	0.968013	0.006309	0.993691
125	*Xenopeltis_unicolor*	0.956362	0.008734	0.991266
126	*Xenosaurus_grandis*	0.947762	0	1
127	*Xinpusaurus_kohi*	0.950614	0.5	0.5
128	*Youngina_capensis*	0.952227	0.346154	0.653846
